# Osteosynthesis of Three- and Four-Part Proximal Humerus Fractures in Elderly Patients Using Locking Plates and Synthetic Bone Grafts: A Clinical and Radiographic Evaluation

**DOI:** 10.7759/cureus.77531

**Published:** 2025-01-16

**Authors:** Antonio Carlos Tenor Júnior, Rafael Segundo Ferreira Neves, Rômulo Brasil Filho, Jorge Assunção, Mauro E Gracitelli, Eduardo A Malavolta

**Affiliations:** 1 Orthopedics and Traumatology, Shoulder and Elbow Group, Instituto de Assistência Médica ao Servidor Público Estadual, São Paulo, BRA; 2 Orthopedics and Traumatology, Shoulder and Elbow Group, Instituto de Assistência Médica ao Servidor Público Estadual, Sao Paulo, BRA; 3 Orthopedics and Traumatology, Shoulder and Elbow Group, Hospital das Clínicas da Faculdade de Medicina da Universidade de São Paulo, São Paulo, BRA; 4 Orthopedics - Shoulder and Elbow, HCor - Hospital do Coração, São Paulo, BRA

**Keywords:** aged, bone cement, bone plates, bone substitutes, calcium sulfate, clinical study, internal fixation of fractures, shoulder fractures, treatment outcome

## Abstract

Objectives: To evaluate clinical and radiographic outcomes and complications of three- and four-part proximal humerus fractures (PHF) in elderly patients treated with angular locking plate (LP) combined with calcium sulfate (CaSO_4_) paste graft.

Materials and methods: A prospective case series evaluating patients aged ≥ 60 years with three- or four-part PHF treated with LP and 10 ml of CaSO^4 ^paste graft. Primary outcome was clinical evaluation using the Constant-Murley (CM) score at 12 months. Secondary outcomes included scores from the University of California, Los Angeles (UCLA), American Shoulder and Elbow Surgeons (ASES), Visual Analog Scale (VAS), Disabilities of the Arm, Shoulder and Elbow (DASH), bilateral active range of motion (ROM), abduction strength, Constant Relative Index (CRI), radiographic findings, and complications.

Results: Thirty patients were evaluated. At 12 months, the mean scores were: 67.7 (CM), 30.5 (UCLA), 86.5 (ASES), 0.7 (VAS), and 18.9 (DASH). The mean active ROM was 121° (flexion), 109° (abduction), 53° (external rotation), and thumb-T12, with abduction strength of 5.7 kg. The mean head-shaft angle (HSA) on the first postoperative day was 133°, with minimal change to 129° at 12 months, and the mean humeral height (HH) remained unchanged (11 mm). There were five complications in four patients (13.3%): three cases of severe varus collapse (difference between the neck-shaft angle (NSA) on the first postoperative day and the 12th month postoperative > 20°), and two cases of cutout. Two reoperations (6.0%) were performed to remove screws that had penetrated the joint.

Conclusion: Clinical and radiographic outcomes of three- or four-part PHF in elderly patients treated with LP and CaSO_4_ paste graft are satisfactory, with relatively low complications and reoperation rates.

## Introduction

Proximal humerus fractures (PHF) are the third most common fractures in the elderly, often associated with osteoporosis and low-energy trauma [1,2]. Most of these fractures are minimally displaced, warranting non-surgical treatment, with surgery reserved for displaced and unstable fractures [3].

Although some studies have shown that surgical treatment outcomes for displaced fractures are not superior to non-surgical treatment in the elderly, both approaches present complications [4]. There is a lack of evidence on whether certain subgroups benefit from surgical intervention for displaced PHFs, and the best approach should be individualized [4,5].

Among surgical treatment options, open reduction and internal fixation (ORIF) with locking plate (LP) is the most commonly used [1,6]. However, due to bone fragility, associated with medial metaphyseal comminution and impaction of the spongy bone of the humeral head, complication rates such as varus collapse and cutout are high, ranging from 6% to 44% [3,7-9].

Techniques to reduce these complications include the use of locked cephalic screws directed to the inferomedial quadrant of the humeral head and bone grafts, autografts, allografts, or synthetic bone substitutes (SBS), either structured or unstructured, for medial metaphyseal support and/or filling of the humeral head defect [1,6,10-12].

The aims of this study were to evaluate clinical and radiographic outcomes and complications of three- or four-part PHF osteosynthesis in elderly patients with LP combined with calcium sulfate (CaSO_4_) paste SBS with a minimum follow-up of one year. Our hypothesis is that the graft association provides good outcomes and relatively low complications and reoperations rates.

## Materials and methods

Study design and participants

This is a prospective case series study conducted at a tertiary hospital. The study began in June 2021, after it was approved by the Institutional Review Board of the Instituto de Assistência Médica ao Servidor Público Estadual (approval 47205821.7.0000.5463), and concluded in October 2024, when the last patient was assessed at 12 months postoperatively. All participants or their legal representatives signed informed consent forms.

Inclusion criteria were elderly patients (age ≥ 60 years); PHF with complete fracture in the surgical neck classified as three or four parts according to modified Neer criteria [13] (displacement of tuberosities > 0.5 cm, angular displacement between the head segment and the humeral shaft > 20° in varus or valgus) [14,15], contact between the shaft and head segment of the humerus < 50% [16], and a maximum time from trauma of 14 days.

Non-inclusion criteria were refusal to sign informed consent; fracture-dislocations [7]; pathological fractures (except osteoporosis); open fractures; associated fractures; fragmentation of the humeral head articular surface or articular fracture line with displacement > 2 mm; inability to understand the questionnaires; shoulder infections; nerve injuries; previous shoulder surgeries; smoking; patients who refused surgical recommendation; any condition of the contralateral shoulder; and high surgical risk (ASA 3).

Criteria for exclusion were inability to perform osteosynthesis detected during the surgery time (conversion to arthroplasty, which in this study accounted for 10% of excluded patients); irreparable rotator cuff injuries detected during the surgery time; and loss to follow-up before completing one year of postoperative care (corresponding to 10% of excluded patients).

Preoperative evaluation

**Figure 1:**
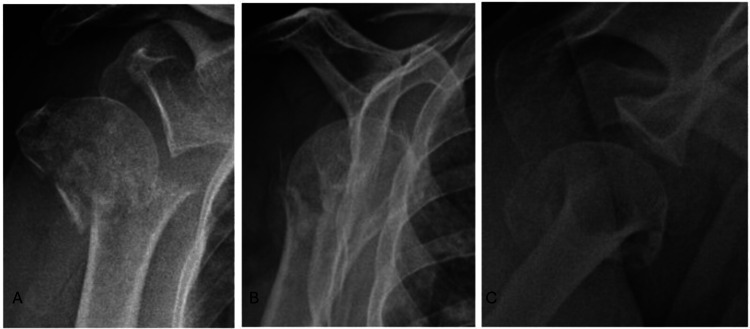
Preoperative radiographic images of the patient in the following views: true anteroposterior (A), scapular profile (B), and Velpau axillary (C) observing the fracture lines in the proximal humerus

**Figure 2:**
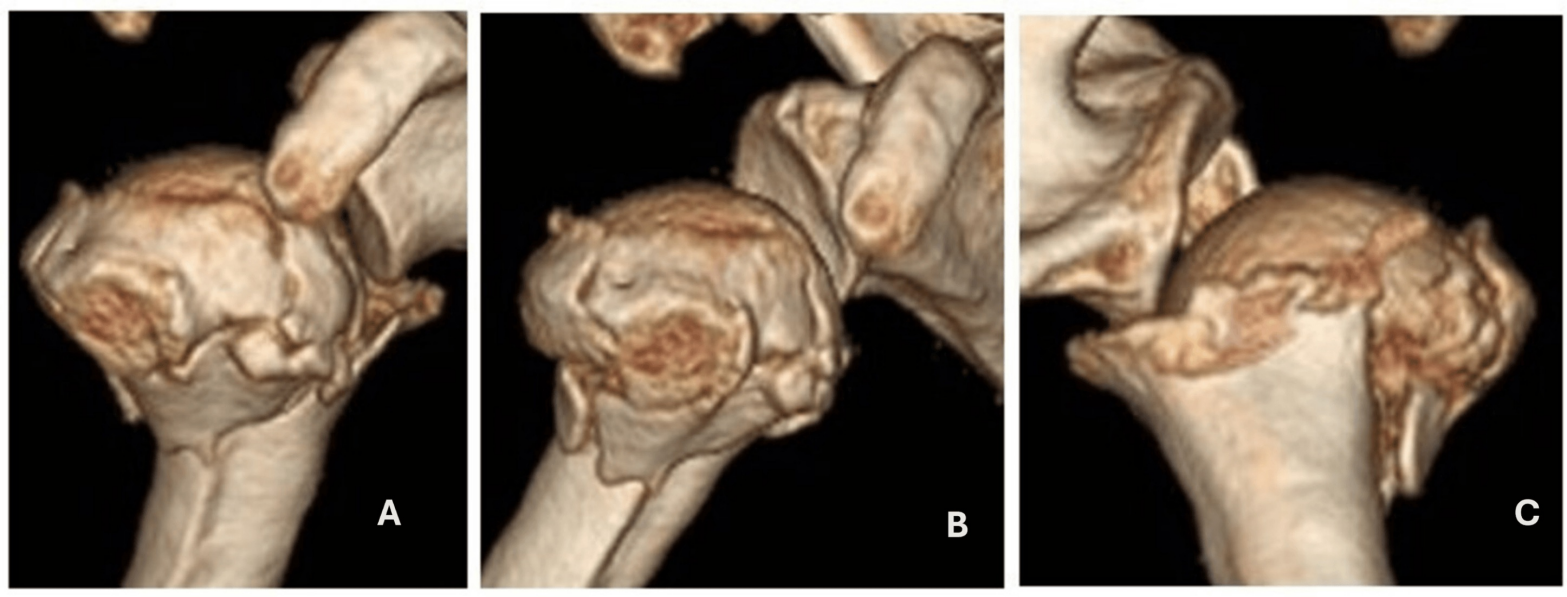
Computed tomography in 3D reconstruction with anterior view (A), anterolateral view (B), and posterior view (C), providing a better understanding of the fracture line.

Radiographs were taken in true anteroposterior view with neutral shoulder rotation, scapular profile, and Velpeau axillary views, as well as computed tomography with flat and three-dimensional reconstruction (Figures 1, 2). Images were analyzed by two independent shoulder surgeons with over 14 years of experience and classified according to modified Neer, Hertel, and Resh criteria [13-15,17].

Deviations of the tuberosity and shaft relative to the humeral head, the size of the metaphyseal fragment of the humeral head, and the presence of metaphyseal comminution were measured. In the true anteroposterior radiograph with neutral shoulder rotation, the head-shaft angle (HSA) was measured, formed by the intersection of the line passing through the center of the humeral head perpendicular to the articular surface line and the bisector of the shaft axis [18,19] of both shoulders of each patient. A third independent evaluator with the same expertise was consulted in case of disagreement. Classifications were confirmed intraoperatively.

Interventions

All surgeries were performed by the lead researcher, who has 18 years of experience in Shoulder and Elbow Surgery, under general anesthesia and interscalene block of the brachial plexus, in the beach chair position (supine with Trendelenburg and about 20° knee flexion, 45° trunk elevation) and 10° lateral decubitus opposite to the operated side, with a C arm fluoroscopy X-ray imaging device positioned posterior to the operating table for image acquisition. An anatomical LP for proximal humerus made of surgical steel with three holes for the shaft (DePuy-Synthes, Solothurn, Switzerland) was used.

**Figure 3:**
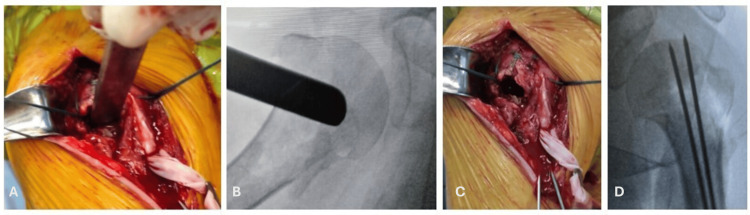
Intraoperative photographic image showing reduction maneuver of an impacted proximal humeral fracture (PHF) by articular surface elevation (A); intraoperative radiographic image showing fluoroscopy control of the reduction maneuver (B); intraoperative photographic image observing void formed beneath the humeral head (C) and intraoperative radiographic image observing PHF provisional fixation with two number two anterograde Kirschner wires (D).

**Figure 4:**
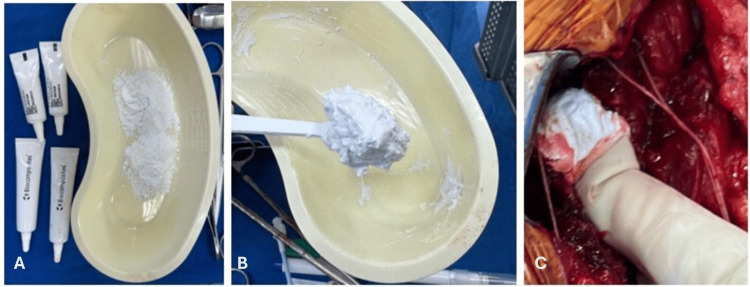
The images illustrate the preparation and application of CaSO4 paste (synthetic bone substitute (SBS)). Photographic image showing calcium sulfate powder (CaSO4 SBS paste precursor) before mixing (A); paste formed after mixing calcium sulfate (B); application of the mixture into the bone void (C).

Through a deltopectoral approach, greater and lesser tuberosities were repaired with high-strength non-absorbable sutures, opened “like a book” for visualization of the articular surface, which was reduced using a Farabeuf retractor or a Lambotte osteotome, with the rotator cuff as the superior limit, and temporarily fixed with two anterograde 2 mm Kirschner wires (Figure 3). The cavity defect between the metaphysis and humeral head was manually filled with 10 ml of lyophilized CaSO_4_ SBS paste, for spongy bone, Osteoset™ (WRIGHT Medical-STRYKER, Memphis, TN, USA) or STIMULAN™ (Biocomposites, Keele, UK) (Figure 4) [20]. After CaSO_4_ setting time (three to five minutes) [6], tuberosities were reduced and secured with previously repaired suture materials. The LP was positioned on the lateral surface of the proximal humerus, aligned with the long axis of its shaft, between 5 and 10 mm posterior to the bicipital groove and distal to the top of the greater tuberosity [1], and fixed with three bicortical screws in the shaft (one cortical and two locking) and six unicortical locking screws in the humeral head, about 0.5 cm from the articular cartilage, with intraoperative radiographic control. Two calcar screws for the medial column [10] were used in all cases. The tuberosities were then fixed to the LP with non-absorbable sutures previously repaired.

Postoperative course

Patients used a sling for six weeks. Rehabilitation, guided by a physical therapist, began on the first postoperative (PO) day with active elbow, wrist, and hand movements and Codman pendulum exercises. At four weeks PO, passive self-assisted exercises, in a supine position, including elevation, lateral rotation, abduction, and standing, extension, and medial rotation, were started. At six weeks PO, active exercises, using only the weight of the limb, were introduced to gradually improve shoulder range of motion (ROM), along with isometric exercises to strengthen the biceps, triceps brachii, trapezius, and rhomboid muscles. The final stage of rehabilitation involved resistance exercises to progressively strengthen the deltoid, rotator cuff, and pectoral muscles, starting at 12 weeks and continuing up to 12 months PO.

Clinical results 

To assess functional outcomes at three, six, and 12 months PO, the following scores were used: Constant-Murley (CM) [21], University of California at Los Angeles (UCLA) [20], American Shoulder and Elbow Surgeons (ASES) [22], Visual Analogue Scale (VAS) for pain, and Disabilities of the Arm, Shoulder, and Hand (DASH) [23].

The Constant and Murley scale, translated and culturally adapted to the Portuguese language by Barreto et al. [20], assigns scores for pain (0 to 15 points), activities of daily living (0 to 20 points), range of movements (0 to 40 points) and strength (0 to 25 points). The maximum score is 100 points, with 86 to 100 points classified as excellent, 71 to 85 points as fair and 0 to 55 points as poor. The Individual Relative Constant (CRI) scale is the percentage obtained by the quotient of the absolute value of the Constant and Murley scale in the affected shoulder by the absolute value in the contralateral one, classified as excellent (90% to 100%), good (80% to 89%), satisfactory (60% to 79%) or poor (< 60%). 

The UCLA scale modified by Ellman, translated and culturally adapted to the Portuguese language by Oku et al. [21], assigns scores for pain (0 to 10 points), function (0 to 10 points), range of active frontal flexion (0 to 5 points), active frontal flexion strength (0 to 5 points) and patient satisfaction (0 to 5 points) and has a maximum score of 35 points, with 34 to 35 points classified as excellent, 28 to 33 points as good, 21 to 27 points as fair and 0 to 20 points as bad.

The ASES scale, translated and culturally adapted to the Portuguese language by Knaut et al. [22], presents an item related to pain, applied through a 10 cm VAS (in which 0 points represent no pain and 10 points, the worst pain possible) and another item related to the function, which consists of 10 questions about daily activities classified from 0 (unable) to 3 (no difficulty). The score obtained in the pain item is multiplied by 5, totaling a maximum value of 50 points and the score obtained in the function item is multiplied by 5/3, totaling a maximum value of 50 points. The final score is obtained by adding the values ​​of items related to pain and function, presenting a maximum value of 100 points, with 91 to 100 points classified as excellent, 81 to 90 points as good, 61 to 80 points as regular and 0 to 60 points as poor.

The DASH questionnaire, translated and adapted into Portuguese by Orfale et al. [23], contains 30 multiple-choice questions that assess the presence of symptoms, function of the upper limbs and aspects of the patient's social life. The score varies from 0 to 100 points, with 0 to 20 points classified as no disability, 21 to 40 points as mild disability, 41 to 60 points.

Bilateral active ROM was measured using a universal goniometer in a standing position for forward flexion, abduction, lateral rotation, and medial rotation (the highest level of the vertebral spinous processes reached by the back of the hand) [1]. Bilateral abduction strength was measured using a Black & Decker BP50 digital dynamometer (Shenzhen, China), and the difference between the averages of three consecutive peak isometric force measurements was calculated. The CM score was also applied to the contralateral shoulder at 12 months PO to calculate the Individual Relative Constant (IRC), categorized as excellent (90%-100%), good (80%-89%), satisfactory (60%-79%), or poor (<60%). Evaluations were conducted by Shoulder and Elbow Surgeons with a minimum of 14 years of experience.

Radiographic parameters

**Figure 5:**
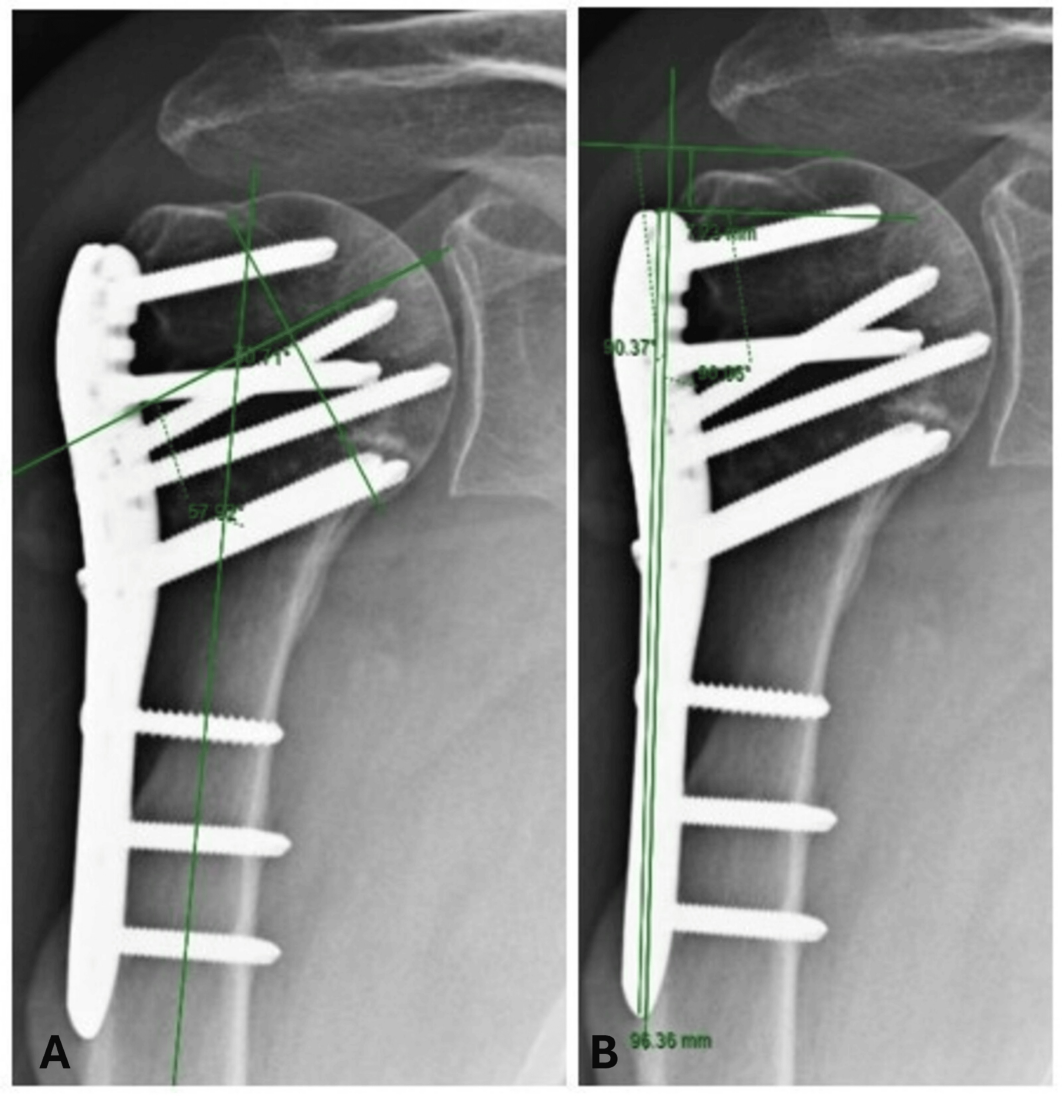
Postoperative Radiographs: Radiographic image of the postoperative follow-up after calculation of the head-shaft angle (HAS) (A) and calculation of humeral height (HH) (B).

Radiographs in true anteroposterior views with neutral rotation of fractured and healthy shoulders, as well as scapular profile and Velpeau axillary views on the fractured side, were taken on the first day, and at six weeks, three months, six months, and 12 months PO. Radiographs were always taken on the same day of the week by a trained radiology team and evaluated by an assistant physician with expertise in Shoulder Surgery, with at least 14 years of experience in Shoulder and Elbow Surgery. The evaluated parameters included the neck-shaft angle (NSA) [18,19] and humeral height (HH) [24], measured as the distance between the apex of the plate and the top of the humeral head in the true anteroposterior view of the shoulder in neutral rotation (Figure 5) [24]. In all three views, the presence or absence of consolidation, defined as the presence of bony bridging (callus or bone trabeculation) in three out of four cortices and obliteration of the fracture line between segments [1], as well as the presence of complications [11,16], were recorded. To correct for magnification in the measured HH on the image and obtain the true HH value, the length of the plate in cm was measured in the true anteroposterior view in neutral rotation, divided by 9 cm (the actual LP length), and the resulting ratio was multiplied by the HH measured on the image [24]. The Carestream Health system (Rochester, NY, USA) was used for image storage and measurements.

Complications

Complications, assessed as binary categorical variables (present or absent), included poor reduction of the tuberosities (displacement > 1 cm on the first PO day), poor reduction of the humeral head (variation between the operated shoulder on the first PO day and the contralateral one > 20°), tuberosities loss of the reduction (displacement > 1 cm after first PO day), collapse of the humeral head in valgus (difference between the NSA on the 12th month and the PO first day PO > 20°) or varus (difference between the NSA on the first PO day and the 12th month PO > 20°) [10,14,19], cutout (penetration of the cephalic screws into the joint) [11], osteonecrosis (ON), pseudarthrosis, lack of consolidation progression [1] over three consecutive months, infection, surgical hardware failure, shoulder stiffness (symptomatic or limiting range of motion starting from the sixth month postoperatively and no improvement after a four-month interval) [1], surgical-related clinical complications.

Statistical analysis

The sample size calculation was based on the Constant and Murley scale for a population like that of our study. In the article by Zhao et al., “Comparison of the Effects of Proximal Humeral Internal Locking System (PHILOS) Alone and PHILOS Combined with Fibular Allograft in the Treatment of Neer Three- or Four-part Proximal Humerus Fractures in the Elderly”, there was a difference of approximately 6 points between two groups of elderly patients with three- or four-part proximal humeral fractures, according to this scale. To calculate the sample size, we assumed a minimum significant difference of 6 points, with a standard deviation of 8 points. To achieve tests with 5% significance and 80% power, at least 29 patients were needed. Therefore, the 30 patients evaluated in this study exceeded the minimum required. 

Initially, all variables were analyzed descriptively. Continuous data are presented as means and standard deviations, and categorical data as absolute numbers and percentages. Continuous variables were analyzed using the Friedman test and Wilcoxon comparisons, with Bonferroni correction. The total number of complications was analyzed considering the number of possible occurrences. The significance level was set at P < 0.05. Statistical analysis was conducted using Python version 3.0 (Python Software Foundation). Data manipulation was performed with the pandas library (version 1.5.3), and statistical calculations were done with the scipy library (version 1.10.1).

## Results

Participant flow

From June 2021 to October 2023, 410 patients were assessed for eligibility, of which 377 (92%) did not meet the inclusion criteria. The most frequent reasons for non-inclusion were two-part PHF, fractures without displacement, age < 60 years, associated fractures, and refusal of surgical indication. A total of 33 patients were included, and three (10%) were lost to follow-up, leaving 30 patients included in the study. The mean age of the patients was 70.30 ± 6.8 years, 90% were female, and the predominant mechanism of injury in 28 of the 30 patients (93%) was a fall from standing height. The baseline characteristics of the patients included in the study are summarized in Table 1.

**Table 1:** Age, trauma mechanism, classification, and deviation of the patients included in the study.

Baseline Characteristics of Patients	Included
	(N=30)
Age	70.3 ± 6.8
Female	27 (90%)
Male	3 (10%)
Machanism of injury	
Standing fall	28 (93%)
Others	2 (7%)
Neer classification	
3 parts	16 (53%)
4 parts	14 (47%)
Displacement in the coronal plane	
Varus	16 (53%)
Valgus	14 (47%)
Medial mataphyseal cominution	20 (66%)

Clinical results

At 12 months, the mean scores were CM 67.6, UCLA 30.5, ASES 86.5, VAS 0.7, and DASH 18.9. The CRI was 86%, and the results were classified as excellent in 3%, good in 15%, and satisfactory in 45%, totaling 73% of patients. The mean values for forward flexion, abduction, external rotation, and internal rotation were 121.3°, 109.5°, 53.2°, and thumb-T12, respectively. The mean abduction strength in the healthy shoulder was 7.6 kg and in the operated shoulder, 5.7 kg. Friedman and Wilcoxon tests with Bonferroni correction for CM, UCLA, ASES, DASH, and VAS functional scores, as well as for range of motion (forward flexion, abduction, and external and internal rotations) over time showed statistically significant differences between the three-, six-, and 12-month postoperative periods. At 12 months, 97% of patients were satisfied with the treatment (Tables 2, 3).

**Table 2:** Clinical scores in the evaluation of the postoperative follow-up of the patients in the study. CM Constant-Murley UCLA University of California at Los Angeles ASES American Shoulder and Elbow Surgeons DASH Disabilities of the Arm, Shoulder, and Hand VAS Visual Analogue Scale *: Mean ± standard deviation **: Friedman test values A: statistically significant in all comparisons (p<0.001 Wilcoxon test with Bonferroni correction B: statistically significant in all comparisons (p<0.05 t-test)

	PATIENTS*	P VALUE
CONSTANT-MURLEY		p<0.001**A
3 MONTHS	35.3 ± 15.1	
6 MONTHS	50.1 ± 16.8	
12 MONTHS	67.6 ± 13.3	
UCLA		p<0.001**A
3 MONTHS	20.1 ± 6.3	
6 MONTHS	25.1 ± 6.4	
12 MONTHS	30.5 ± 4.4	
ASES		p<0.001**A
3 MONTHS	55.7 ± 21.4	
6 MONTHS	69.9 ± 21.9	
12 MONTHS	86.5 ± 17.0	
DASH		p<0.001**A
3 MONTHS	41.6 ± 18.3	
6 MONTHS	32.7 ± 21.2	
12 MONTHS	18.9 ± 15.4	

**Table 3:** Evaluation of the postoperative follow-up of the patients' range of motion. *: Mean ± standard deviation **: Friedman test values A: statistically significant in all comparisons (p<0.001 Wilcoxon test with Bonferroni correction)

	PATIENTS*	P VALUE
FORWARD FLEXION		p<0.001**A
3 MONTHS	72° ± 24°	
6 MONTHS	99° ± 30°	
12 MONTHS	121° ± 29°	
ABDUCTION		p<0.001**A
3 MONTHS	66° ± 22°	
6 MONTHS	89° ± 32°	
12 MONTHS	109° ± 31°	
EXTERNAL ROTATION		p<0.001**A
3 MONTHS	13° ± 10°	
6 MONTHS	30° ± 19°	
12 MONTHS	53° ± 20°	
INTERNAL ROTATION		p<0.001**A
3 MONTHS	17 ± 2	
6 MONTHS	14 ± 3	
12 MONTHS	11 ± 3	

Radiographic parameters

The values for NSA and HH on the first day and at 12 months postoperatively are shown in Table 4. There was 100% consolidation between six weeks and three months PO. Friedman test results indicate a statistically significant difference for NSA, but no significant difference for HH between assessment times. However, Wilcoxon comparisons, after Bonferroni correction, showed that most time pairs did not exhibit significant differences for NSA, except between the first day and 12th months postoperatively, and confirmed the absence of a statistically significant difference for HH.

**Table 4:** Radiographic evaluation of the postoperative follow-up of the patients. *: Mean ± standard deviation **: Friedman test values C: statistically significant only in the comparisons at six weeks and 12 months (p>0.05 Wilcoxon test with Bonferroni correction) X: no statistical significance after Wilcoxon tests with Bonferroni correction

	PATIENTS*	P VALUE
HEAD-SHAFT ANGLE		P<0.05**C
FIRST DAY POSTOPERATIVELY	133.3° ± 9.4°	
6 WEEKS	131.0° ± 11.8°	
3 MONTHS	131.8° ​​​​​​​ ± 10.9° ​​​​​​​	
6 MONTHS	131.5° ​​​​​​​ ± 10.9° ​​​​​​​	
12 MONTHS	128.8° ​​​​​​​ ± 11.7° ​​​​​​​	
HUMERAL HEIGHT		p=0.390**X
FIRST DAY POSTOPERATIVELY	11.1 mm ​​​​​​​± 4.1 ​​​​mm	
6 WEEKS	10.7 mm ± 3.5 mm	
3 MONTHS	11.3 mm ± 4.1 mm	
6 MONTHS	10.8 mm ± 3.8 mm	
12 MONTHS	10.8 mm ± 3.6 mm	

Among the 30 cases analyzed, 25 (73.3%) showed no postoperative angular deviation greater than 5°, with an average variation of -1.56°. Varus deviation was observed in seven cases (23.3%), with an average variation of -12.5°. Only one case presented valgus deviation, with a variation of 14.0° (Table 5).

**Table 5:** Evaluation of the residual deviation of the patients.

Cohort	Number of patients	(%)	Average variation
No angular deviation	22	73.3%	-1.56°
Varus deviation	7	23.3%	-12.5°
Valgus deviation	1	3.3%	14.0°

Complications

There were five complications in four patients (13.3%) and one death at nine months postoperatively due to clinical causes unrelated to the intervention. Among the complications, there were three cases of collapses in varus and two cases of cutout. There were two reoperations to remove synthesis material (6.6%) in patients who experienced cutout, but there was no need for implant replacement or conversion to shoulder arthroplasty (Table 6). The mean follow-up period for complications was 12 months.

**Table 6:** Complications observed during the postoperative follow-up of the patients.

Complications	N (%)
Total	4 (13.3%)
varus collapse	2 (6.6.%)
Cutout	2 (6.6%)
Reoperations (remove synthesis material for cutout)	2 (6.6%)
Death (unrelated to surgery)	1 (3.3%)

## Discussion

Our findings confirm the hypothesis that CaSO_4_ paste grafts, used for augmentation with LP osteosynthesis in complex PHF in elderly patients, result in good functional satisfactory outcomes and relatively low rates of complications and reoperations. There was a significant gradual improvement in all functional scores through the intermediate evaluations up to 12 months postoperatively.

The results are consistent with those described by Marongiu et al. [6] in a systematic review of synthetic grafts, as well as with studies by Liu et al. [25], using CaSO_4_, and Egol et al. [24], using Ca_^3^_(PO_4_)_2_, which reported better functional outcomes and lower complication rates, such as collapse and cutout, in the graft-associated groups.

We achieved scores of 67.6 and 18.9 on the CM and DASH scales, respectively; results slightly higher than those reported by Somasundaran et al. [26] (64 and 16.2 points) for complex shoulder fractures treated with CaSO_4_ paste grafts. Similarly to Robinson et al. [18], we achieved 100% consolidation in severely impacted shoulder fractures treated with Ca_3_(PO_4_)_2_ paste grafts, few severe losses of reduction and satisfactory functional results, without any osteonecrosis.

Our patients' average ROMs were 121.3° of anterior flexion, 109.5° of abduction, 53.2° of lateral rotation, and back of the hand - T11 of medial rotation. These results surpass those reported by Sproul et al. [8], who found 98° of flexion and 103° of abduction in patients with a 10-year younger average age, treated for PHF in two, three, or four parts fixed with LP without grafts.

The average HSA and HH on the first day and at 12 months postoperatively were 134° and 11.5 mm, and 129° and 11 mm, respectively, while contralateral HSA was 138°, and 75.8% among our patients showed no postoperative angular deviation greater than 5°. These results indicate a good reduction achieved and maintained until consolidation. These outcomes are similar to those reported by Egol et al. [24], who observed less variation in HSA and HH with Ca_3_(PO_4_)_2_ grafts, and by Seebach et al. [19], who noted an HSA average of 140° with variation < 20° in elderly patients with complex PHF treated with LP fixation and β-tricalcium phosphate granules grafts.

Our complications and reoperations rates (13.3% and 6.6%, respectively) were significantly lower than those reported in three systematic reviews of clinical studies on PHF treated with LP without grafts, which included fractures in two, three, or four parts and younger patients, with lower average ages. Brorson et al. [9] reported up to 16% loss of reduction, 20% cutout, 44% reoperations, and 33% ON; Thanasas et al. [7] reported 11.6% cutout, 13.7% reoperations, and 7.9% ON; and Sproul et al. [8] reported 49% complications (16% varus collapse, 19% ON, 8% cutout, 6% subacromial impingement, and 4% infection) and 14% reoperations.

Rangan et al. [4], in a randomized controlled trial, reported 18.4% complications with conservative treatment of complex shoulder fractures, with 16% classified as major complications leading to an 8.8% surgical rate, including some reverse shoulder arthroplasties. Our rates of major complications and reoperations were slightly lower and were confined to material removal surgeries.

Suroto et al. [27], in a systematic review and meta-analysis, reported 65.9 points on the CM score, 122.2° of anterior flexion, 105.7° of abduction, and 5.5% reoperation rate with reverse shoulder arthroplasty for treating complex shoulder fractures in a predominantly elderly, female population. Our patients had an average age of 70.3 years, with 87% being female, and our results were similar. However, reverse arthroplasty resulted in a smaller gain in lateral rotation (average of 27.4°), almost 100% less than what we achieved, and the complication rates of reverse arthroplasty, which can reach 32.7%, were approximately 145.4% higher than ours, with the added factor that managing total shoulder arthroplasty complications is more complex.

Due to the impossibility of objectively assessing preoperative functional scores to calculate magnitude and strength of findings, we based it on the human discrimination limit, which equals an effect size of 0.46 units [28]. Therefore, an intervention effect should be considered clinically relevant if > ½ standard deviation [28]. With scores exceeding the discrimination limit at three, six, and 12 months postoperatively, our functional results were both statistically significant and clinically important.

The present study’s limitations include the lack of a control group, follow-up duration, low reproducibility of classification systems [15], and difficulty in obtaining identical radiographs, as shoulder positioning can alter HSA measurements [29].

However, although ON can occur up to two years, most cases occur within the first 12 months, and follow-up for other outcomes is considered sufficient [1].

To improve classification reproducibility, we standardized radiographs, always performed CT scans with reconstruction, had two experienced and independent examiners classify the fractures, a third evaluator reviewed disagreements, and classifications were confirmed intraoperatively.

Postoperatively, measurements were taken with the shoulder in neutral rotation at two different times and the average of the measurements was considered. Assunção et al. [29], in a clinical study, found no significant differences in HSA with the shoulder in neutral rotation versus maximum lateral rotation of 30°.

The lead researcher performed all surgeries to avoid performance bias. Claro et al. [30] reported complications and reoperation rates twice as high when patients were operated on by non-specialists in shoulder surgery. Other strengths of the study include the magnitude and strength of findings estimation [28], prospective data collection, inclusion and exclusion criteria to eliminate confounding variables, use of calcar screws and tuberosity sutures in all patients, variables that influence the outcomes [7,10,26].

It is important to highlight that the strengths of the study included the calculation of sample size, the availability of both devices - the locking plate and the reverse prosthesis, sterilized, in all operations - and the assessment of the feasibility of performing osteosynthesis to avoid the bias of inadequate intervention. We also emphasize the selection criteria used to obtain a homogeneous sample and eliminate confounding factors, the prospective data collection to avoid recall bias, and the standardization of the surgical procedure, including the use of inferomedial screws and the tying of the tuberosities to the plate - variables that could influence outcomes - to minimize confounding bias. Furthermore, it is worth noting among the study's strengths the fact that few studies evaluate the outcomes and complications of combining synthetic calcium sulfate grafts with the osteosynthesis of proximal humeral fractures. This technique has also been shown to be reproducible, safe, effective, and does not increase surgical morbidity. However, we also highlight the study's limitations, such as the difficulty in verifying the consolidation of proximal humeral fractures within the first three months after surgery, as well as the fact that all operations were performed at a single center by a single surgeon, which may affect the external validity of the study.

Few clinical studies evaluate the outcomes of CaSO_4_ paste graft augmentation with complex shoulder fractures in osteoporotic bones. We believe our results offer a valuable tool that can influence decision-making in daily practice, as this technique has proven reproducible, safe, and effective, and does not add morbidity to the surgery.

## Conclusions

The use of synthetic CaSO4 bone substitute for augmentation of bone defects, combined with LP osteosynthesis for complex three- or four-part PHFs in elderly patients, resulted in satisfactory clinical and radiographic outcomes, with relatively low rates of complications and reoperations. Future randomized clinical trials will be essential to confirm the potential benefits of this association.
